# Persistent Periorbital Edema as the Initial Manifestation of Probable Systemic Lupus Erythematosus With Suspected Protein-Losing Enteropathy or Lupus Serositis in an Adolescent: A Case Report

**DOI:** 10.7759/cureus.113454

**Published:** 2026-07-27

**Authors:** Rehena Akhter, Shahidul Hassan Mollick, Muhammad Misqatus Saleheen

**Affiliations:** 1 Department of Gastroenterology, Khulna Medical College, Khulna, BGD; 2 Department of Dermatology and Venereology, 250 Bedded General Hospital Khulna, Khulna, BGD

**Keywords:** adolescent, case report, periorbital edema, protein-losing enteropathy, systemic lupus erythematosus

## Abstract

A 16-year-old girl presented with a three-month history of progressive, painless, bilateral periorbital edema without renal, hepatic, thyroid, or allergic etiology. Physical examination revealed bilateral periorbital puffiness without erythema, tenderness, malar rash, synovitis, or peripheral edema. Laboratory studies demonstrated a hemoglobin level of 8.3 g/dL, erythrocyte sedimentation rate of 115 mm/hr, serum albumin at 2.6 g/dL, antinuclear antibody greater than 400 AU/mL (normal: <40), and anti-double-stranded DNA antibody greater than 800 IU/mL. Urinalysis and 24-hour urinary protein excretion (120 mg/day) were normal. Abdominal ultrasonography revealed moderate ascites with normal liver parenchyma, portal vein caliber, and renal architecture. Chest radiography demonstrated no pleural effusion or other cardiopulmonary abnormality. A clinical diagnosis of probable systemic lupus erythematosus was made based on strongly positive autoantibodies, active systemic inflammation, unexplained anemia, and moderate ascites with hypoalbuminemia in the absence of alternative etiologies. The combination of hypoalbuminemia, ascites, and absent proteinuria raised suspicion for protein-losing enteropathy or lupus serositis; however, confirmatory testing was unavailable. The patient received oral prednisolone at 1 mg/kg/day based on a documented body weight of 50 kg. Within seven days, periorbital edema markedly improved. Serum albumin normalized to 3.8 g/dL by three months. This case illustrates that isolated periorbital edema may be an initial manifestation of systemic lupus erythematosus in adolescents, and the presence of hypoalbuminemia with ascites in the absence of proteinuria should prompt consideration of protein-losing enteropathy or serositis in the differential diagnosis. Early immunological evaluation and corticosteroid therapy were associated with favorable clinical and biochemical outcomes.

## Introduction

Systemic lupus erythematosus (SLE) is a chronic autoimmune disease characterized by multisystem involvement and the production of autoantibodies against nuclear and cytoplasmic antigens [1]. Childhood-onset SLE, defined as disease onset before 18 years of age, accounts for approximately 15% to 20% of all SLE cases and is associated with a more aggressive disease course, higher disease activity, and greater cumulative organ damage compared with adult-onset disease [1].

Periorbital edema is a nonspecific clinical finding that may occur in a variety of conditions associated with hypoalbuminemia, including nephrotic syndrome, chronic liver disease, severe malnutrition, protein-losing enteropathy (PLE), and other systemic disorders. In patients with SLE, periorbital edema most commonly results from lupus nephritis or nephrotic syndrome, which affect a substantial proportion of childhood SLE patients during the disease course [2]. However, isolated periorbital edema without evidence of renal involvement is an uncommon presenting feature. Alternative mechanisms include lupus serositis with ascites, increased vascular permeability, and PLE [3,4].

PLE is a rare gastrointestinal complication of SLE characterized by excessive protein loss through the gastrointestinal tract, leading to hypoalbuminemia, edema, and ascites in the absence of proteinuria [5,6]. The estimated prevalence of PLE in SLE remains uncertain due to underdiagnosis and lack of standardized diagnostic criteria, with reported frequencies around 1.9% in some tertiary referral center series [6]. Gastrointestinal symptoms may be minimal or absent, particularly in pediatric populations, which contributes to diagnostic delay [4,6]. The pathophysiology of PLE in SLE remains incompletely understood but may involve immune-mediated mucosal damage, lymphatic obstruction, or increased mucosal permeability [5,6].

Carneiro et al. reported a case of PLE as the presenting feature of SLE in an 18-year-old female, highlighting that gastrointestinal protein loss may precede other systemic features [7]. Al-Khaldi described bilateral orbital edema as a presenting feature of cutaneous lupus erythematosus with negative serology in a 32-year-old female, with potential for later development of SLE with positive serology [3]. Sliman et al. reported a 16-year-old female with bilateral periorbital edema as the sole initial manifestation of SLE who subsequently developed lupus nephritis class II on renal biopsy [8]. These reports collectively suggest that periorbital edema, whether from nephrotic syndrome, serositis, or PLE, may serve as a sentinel sign of underlying lupus.

We report a case of a 16-year-old girl who presented with isolated periorbital edema as the initial manifestation of SLE, with clinical and laboratory findings suggestive of PLE or lupus serositis. This case was prepared following the CARE (CAse REport) guidelines for case reports [9].

## Case presentation

A previously healthy 16-year-old girl presented to the outpatient clinic with a three-month history of progressive, painless, bilateral periorbital edema. The swelling was nonpruritic, nonerythematous, and not associated with visual disturbance, ocular pain, or photophobia. She denied peripheral edema, lip or tongue swelling, dyspnea, fever, arthralgia, skin rash, oral ulcers, alopecia, abdominal pain, diarrhea, constipation, recent weight loss, or loss of appetite. There was no history of recent medication use, allergen exposure, or family history of autoimmune disease.

**Figure 1:**
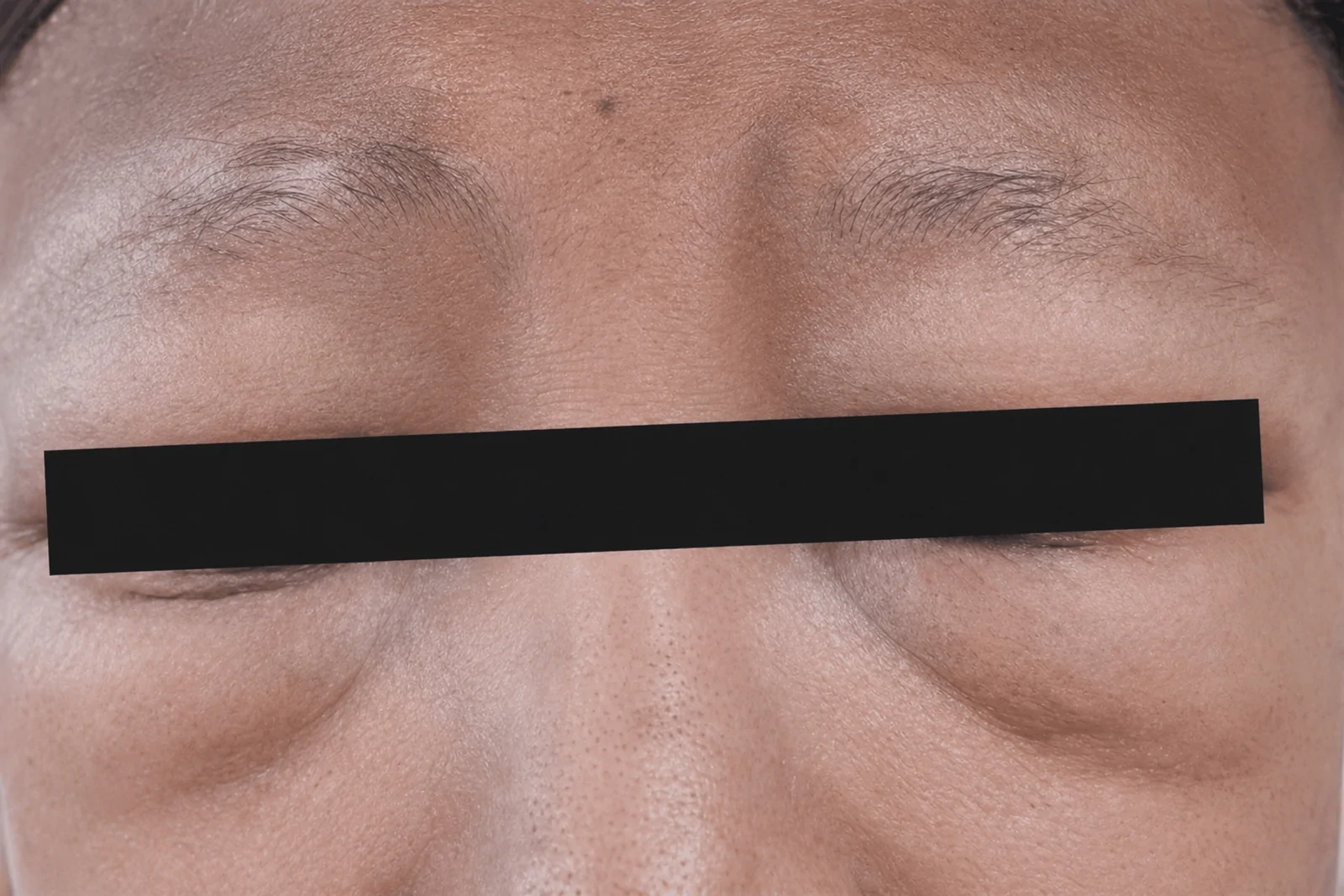
Bilateral periorbital edema at presentation. The patient at initial presentation demonstrating bilateral periorbital edema before initiation of corticosteroid therapy. The eyes have been masked to preserve patient privacy.

On physical examination, vital signs were within normal limits. Bilateral periorbital puffiness was present without erythema, warmth, or tenderness (Figure 1).

There was no malar rash, discoid lesions, synovitis, or peripheral edema. Cardiovascular, respiratory, and abdominal examinations were unremarkable except for shifting dullness suggesting ascites.

Initial laboratory investigations revealed a hemoglobin level of 8.3 g/dL (reference range: 11.5-15.5 g/dL), total leukocyte count of 5,200 cells/µL (reference: 4,000-11,000), platelet count of 214 x 10^3/µL (reference: 150-450), and erythrocyte sedimentation rate of 115 mm/hr (reference: <30). Serum albumin was 2.6 g/dL (reference: 2.8-5.4 g/dL). C-reactive protein (CRP) was elevated at 20 mg/L (reference range: <6 mg/L), consistent with active systemic inflammation. Serum creatinine was 0.6 mg/dL (reference: 0.5-1.3), and liver function tests, including alanine aminotransferase, aspartate aminotransferase, and alkaline phosphatase, were within normal limits. Thyroid-stimulating hormone and electrolytes were also normal. Urinalysis was unremarkable, and 24-hour urinary protein excretion was 120 mg/day (reference: <150), effectively excluding nephrotic-range proteinuria. The patient's key laboratory findings at presentation and three-month follow-up are summarized in Table 1.

**Table 1:** Laboratory parameters at presentation and three-month follow-up. Anti-dsDNA: anti-double-stranded DNA.

Parameter (units)	At presentation	At three-month follow-up	Normal range
Hemoglobin (g/dL)	8.3	11.2	11.5-15.5
Erythrocyte sedimentation rate (mm/hr)	115	35	Less than 30
Serum albumin (g/dL)	2.6	3.8	2.8-5.4
Urinalysis	Normal	Normal	---
24-hour urinary protein (mg/day)	120	Not repeated	Less than 150
Antinuclear antibody (AU/mL)	Greater than 400	Not repeated	Less than 40
Anti-dsDNA antibody (IU/mL)	Greater than 800	Not repeated	<30 IU/mL

Autoimmune serological testing demonstrated an antinuclear antibody level greater than 400 AU/mL (reference range: <40 AU/mL) and an anti-double-stranded DNA antibody level greater than 800 IU/mL (reference range: negative <30 IU/mL, positive ≥30 IU/mL), as reported by the hospital laboratory. Serum complement components C3 and C4 and the direct Coombs test were unavailable at our institution.

**Figure 2:**
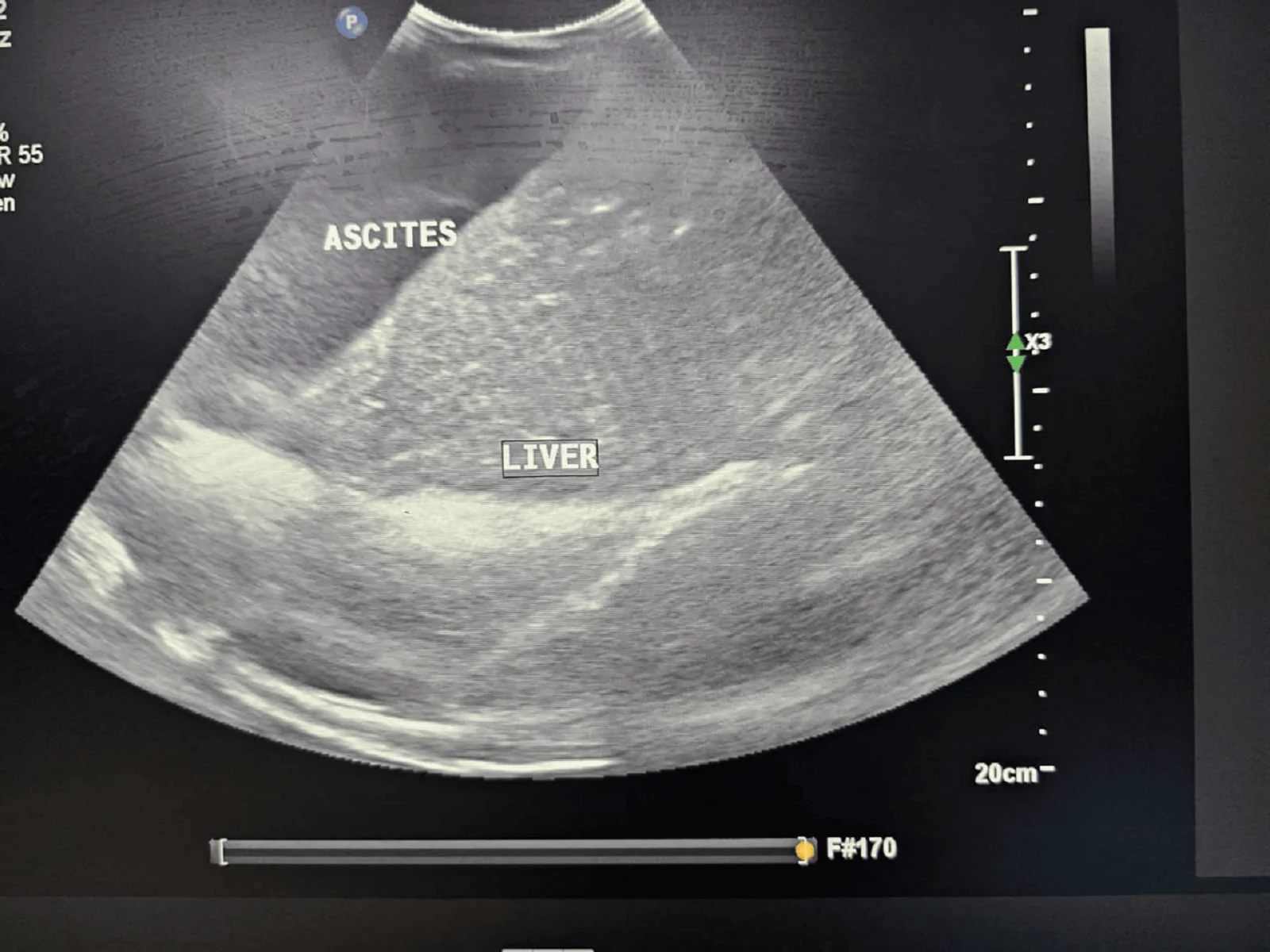
Abdominal ultrasonography showing moderate ascites. Abdominal ultrasonography demonstrating moderate ascites with preserved liver parenchyma, normal portal vein caliber, and normal renal architecture.

Abdominal ultrasonography revealed moderate ascites with normal liver parenchyma, portal vein caliber, and renal architecture (Figure 2).

**Figure 3:**
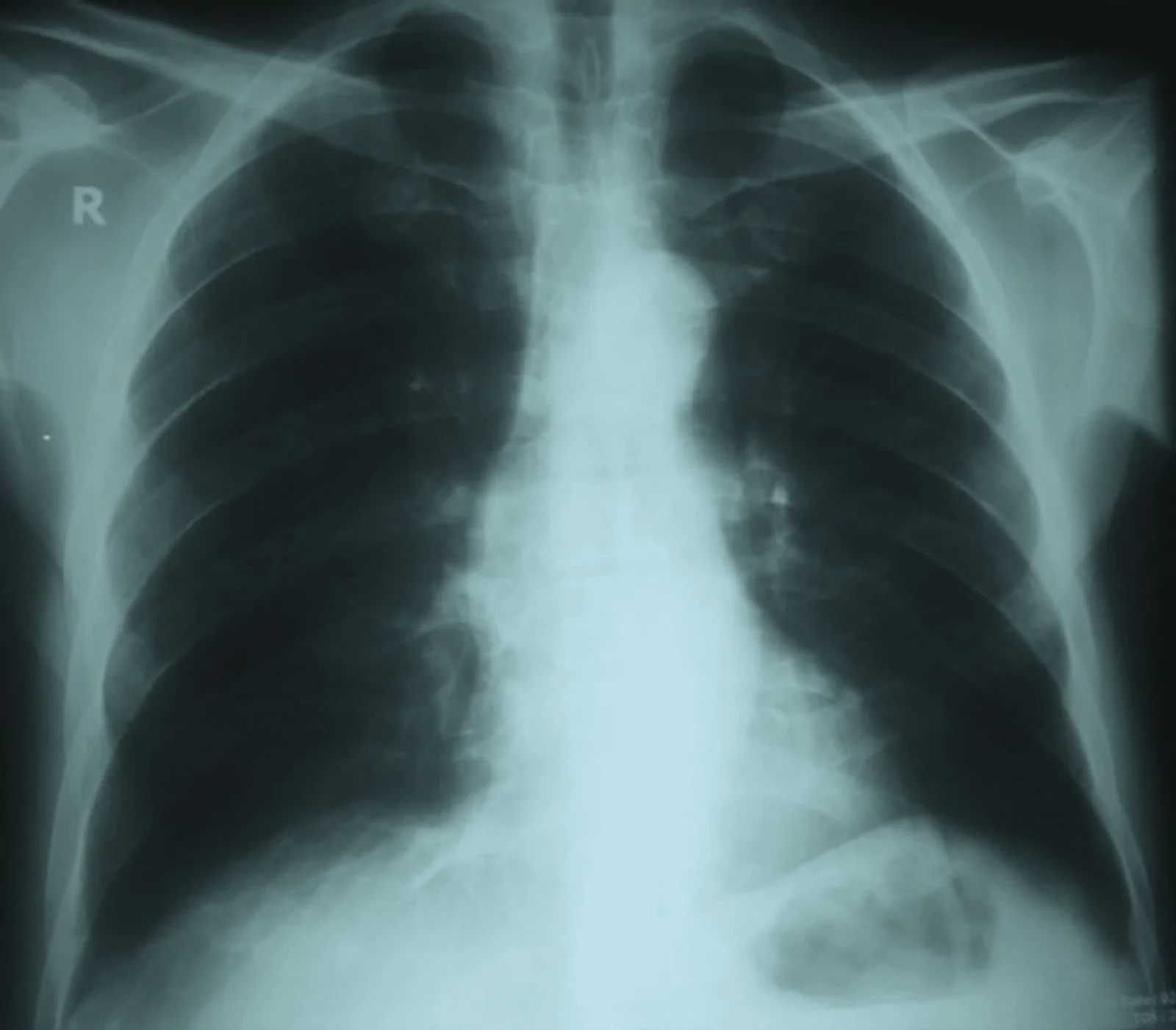
Normal chest radiograph. Chest radiograph demonstrating no significant cardiopulmonary abnormality. The cardiac silhouette is within normal limits, and no focal pulmonary infiltrate, pleural effusion, or other acute abnormality is identified.

Chest radiography showed no significant cardiopulmonary abnormality (Figure 3).

**Figure 4:**
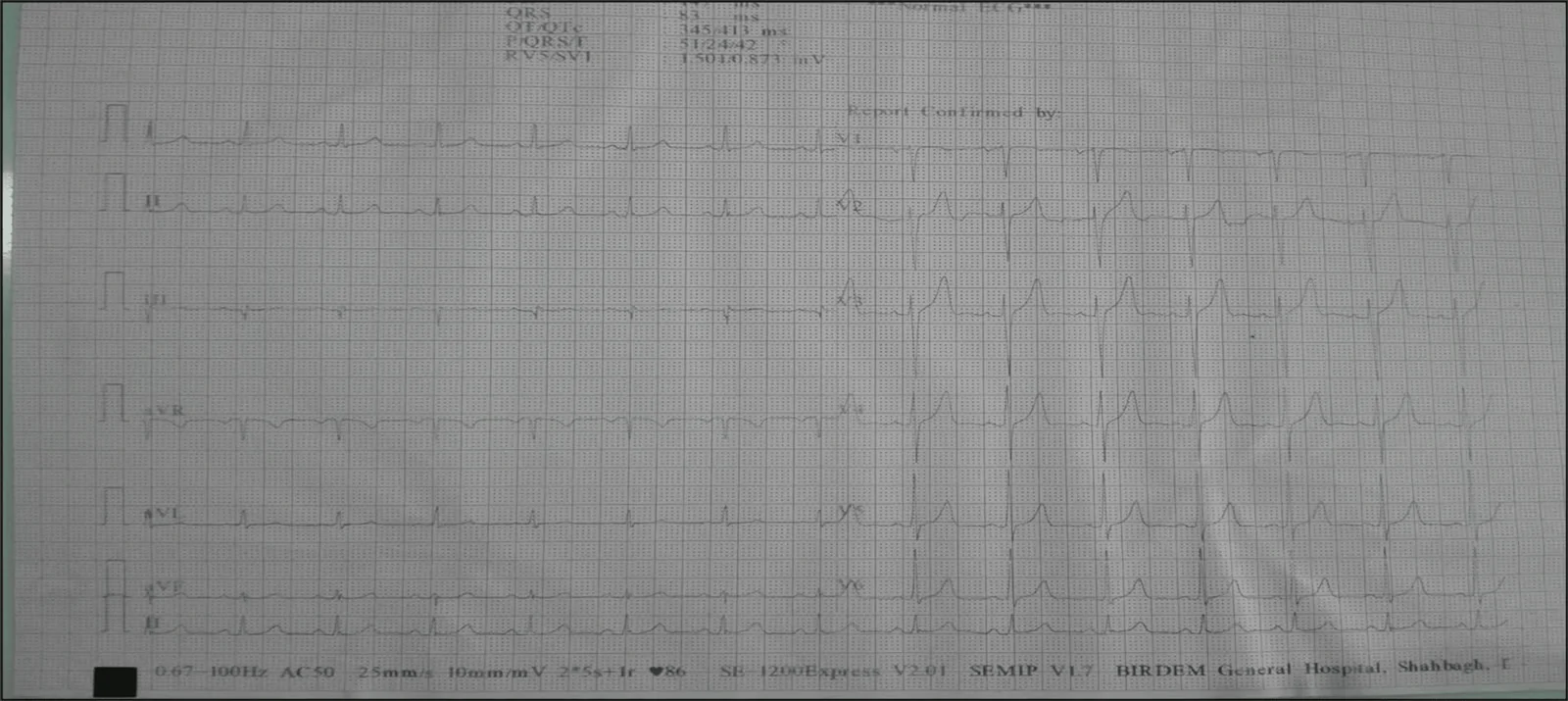
Normal electrocardiogram. Twelve-lead electrocardiogram showing normal sinus rhythm with no significant conduction abnormality or ischemic change.

Serological markers for viral hepatitis were negative. Electrocardiography demonstrated normal sinus rhythm without significant abnormalities (Figure 4).

The differential diagnosis included nephrotic syndrome, chronic liver disease, hypothyroidism, allergic angioedema, and severe malnutrition. Nephrotic syndrome was considered unlikely because urinalysis and 24-hour urinary protein excretion were normal. Chronic liver disease was excluded by normal liver function tests and abdominal ultrasonography. Hypothyroidism was excluded by a normal thyroid-stimulating hormone level, and allergic angioedema was considered unlikely because of the chronic painless course and absence of allergen exposure or urticaria.

A clinical diagnosis of probable SLE was made based on the combination of strongly positive autoantibodies (antinuclear antibody greater than 400 AU/mL, anti-double-stranded DNA antibody greater than 800 IU/mL), active systemic inflammation (erythrocyte sedimentation rate = 115 mm/hr), unexplained anemia (hemoglobin = 8.3 g/dL), and moderate ascites with hypoalbuminemia in the absence of alternative etiologies. Both the 2012 Systemic Lupus International Collaborating Clinics and the 2019 European League Against Rheumatism/American College of Rheumatology classification criteria were considered as supportive frameworks [10,11]. However, formal classification was limited by the unavailability of confirmatory investigations, including serum complement levels, direct Coombs testing, and the fact that peritoneal involvement is not a scored item within the serosal domains of either classification system. The clinical phenotype, autoantibody profile, and response to corticosteroid therapy were consistent with probable SLE, and the diagnosis was made using established clinical judgment.

The constellation of hypoalbuminemia (2.6 g/dL), moderate ascites, and normal urinary protein excretion raised strong suspicion for PLE. Alternative considerations included lupus peritonitis or serositis. Unfortunately, confirmatory investigations for PLE, including fecal alpha-1 antitrypsin clearance, technetium-99m albumin scintigraphy, or upper and lower gastrointestinal endoscopy with biopsy, were unavailable at our center. Paracentesis with fluid analysis was not performed.

**Figure 5:**
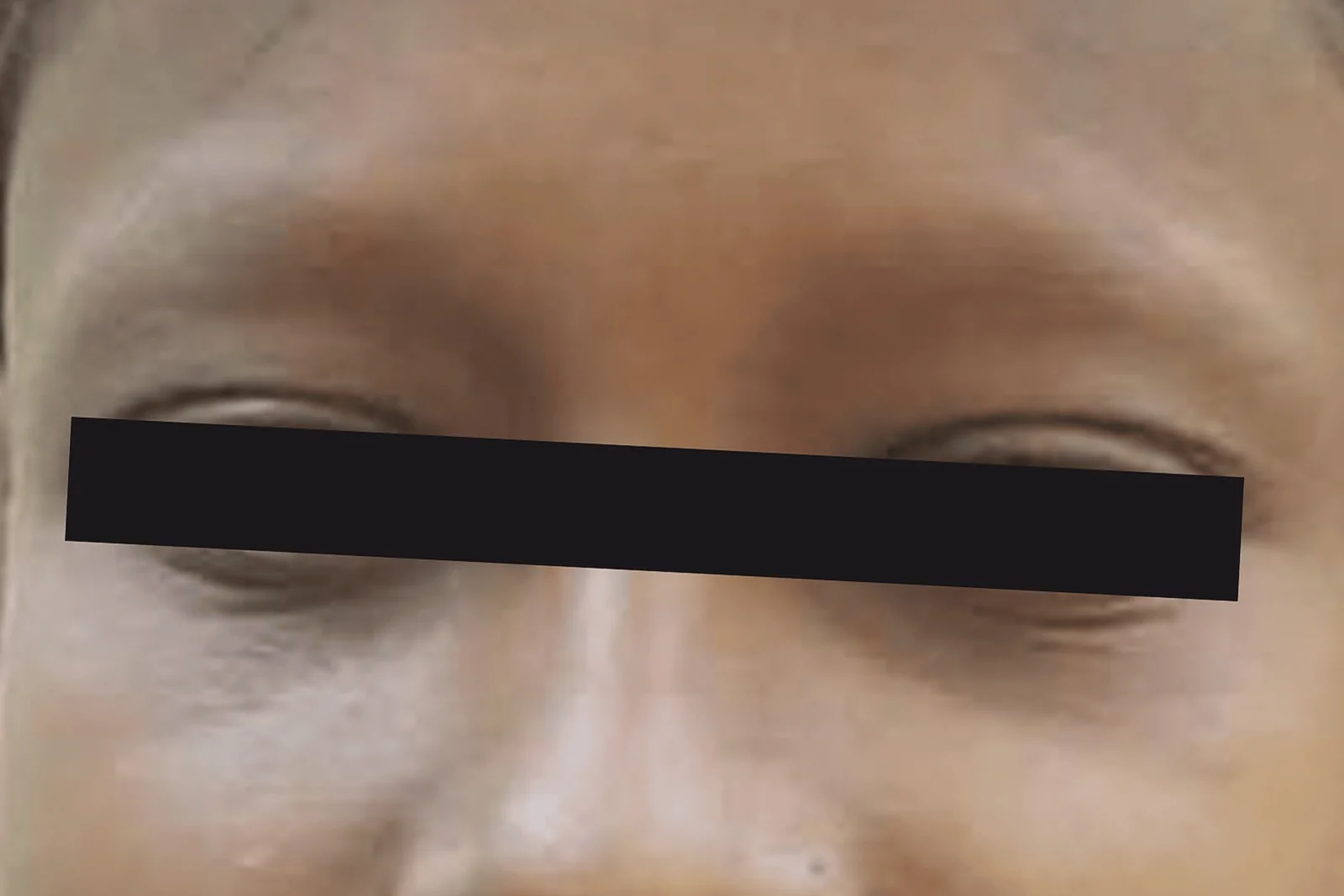
Improvement after prednisolone therapy. Marked reduction of bilateral periorbital edema after seven days of oral prednisolone therapy. The patient's eyes have been masked to preserve privacy.

The patient was initiated on oral prednisolone at 1 mg/kg/day based on a documented body weight of 50 kg. Within seven days of corticosteroid therapy, the periorbital edema demonstrated marked reduction (Figure 5).

The patient was discharged with instructions for gradual ambulation and weekly outpatient follow-up.

At the three-month follow-up evaluation, the patient was asymptomatic with complete resolution of periorbital edema and ascites. Repeat laboratory studies demonstrated a hemoglobin level of 11.2 g/dL (improved from 8.3 g/dL but still below the reference range of 11.5-15.5 g/dL) and serum albumin level of 3.8 g/dL (reference: 2.8-5.4 g/dL). The erythrocyte sedimentation rate had decreased to 35 mm/hr (reference: <30), indicating persistent though improved inflammatory activity. Rheumatology referral was arranged for consideration of steroid-sparing agents and long-term disease monitoring.

Written informed consent for publication, including the use of clinical photographs, was obtained from the patient and her legal guardian. This report was prepared in accordance with the CARE guidelines [9].

## Discussion

Isolated periorbital edema is an uncommon initial manifestation of SLE, particularly in the absence of overt renal involvement. This case highlights important diagnostic considerations for clinicians evaluating adolescents with unexplained edema.

Differential diagnosis of periorbital edema in SLE

The differential diagnosis of periorbital edema in a patient with suspected or confirmed SLE includes lupus nephritis with nephrotic syndrome, lupus serositis with ascites, increased systemic vascular permeability, and PLE [2,4,5]. In the present case, the normal urinalysis and 24-hour urinary protein excretion effectively excluded nephrotic syndrome as the primary mechanism. The presence of moderate ascites on ultrasonography, combined with hypoalbuminemia and normal hepatic and renal function, directed diagnostic consideration toward intra-abdominal protein loss or serositis.

Protein-losing enteropathy versus lupus serositis

Differentiating PLE from lupus peritonitis or serositis in SLE presents a significant clinical challenge, particularly when advanced diagnostic modalities are unavailable. PLE in SLE typically presents with hypoalbuminemia, edema, and ascites without proteinuria, often in the setting of active systemic disease [5,6]. The proposed pathophysiological mechanisms include immune-mediated intestinal mucosal injury with increased permeability, complement activation, and lymphatic dilation or obstruction [4,5]. Lupus serositis, by contrast, involves inflammation of the peritoneal lining with exudative fluid accumulation. The ascitic fluid in serositis typically demonstrates elevated protein content and inflammatory cells, whereas PLE-related ascites is generally transudative with low protein content reflective of hypoalbuminemia. Unfortunately, paracentesis with fluid analysis was not performed in this case, precluding definitive differentiation between these mechanisms.

In this case, the absence of abdominal pain, the presence of significant hypoalbuminemia, and the lack of proteinuria favored PLE as the working diagnosis. However, without paracentesis with fluid analysis, fecal alpha-1 antitrypsin clearance measurement, or scintigraphic studies, definitive differentiation between PLE and lupus serositis was not possible. We have therefore characterized the presentation as "suspected PLE or lupus serositis" to accurately reflect the diagnostic uncertainty.

Comparison with previously reported cases

Several case reports have described PLE as a complication or initial manifestation of SLE. Carneiro et al. reported an 18-year-old female in whom PLE was the presenting feature of SLE, with hypoalbuminemia (1.9 g/dL), ascites, and normal renal function [7]. Chen et al., in a 12-year retrospective series from a Chinese academic center, identified PLE in 1.9% of SLE patients, with the majority presenting with hypoalbuminemia and edema [6]. Chen et al. noted that gastrointestinal symptoms were absent in a substantial proportion of patients with SLE-associated PLE, contributing to diagnostic delay [6]. Similar observations have also been reported among adult patients with SLE-associated PLE. Therefore, the absence of gastrointestinal symptoms should not exclude the diagnosis of SLE-associated PLE in either pediatric or adult patients.

Al-Khaldi et al. described bilateral orbital edema as a presenting feature of cutaneous lupus erythematosus in a 32-year-old female with negative antinuclear antibody (ANA) and anti-double-stranded DNA (anti-dsDNA) antibody tests at presentation, with potential for later development of SLE with positive serology [3]. Sliman et al. reported a 16-year-old female with bilateral periorbital edema as the sole initial manifestation of SLE who subsequently developed lupus nephritis class II on renal biopsy [8]. Notably, that patient had normal serum albumin at presentation and developed proteinuria during follow-up, in contrast to our patient who presented with hypoalbuminemia and ascites without proteinuria.

Our case differs from these previously reported cases in several important respects. First, our patient presented with hypoalbuminemia and ascites without proteinuria, suggesting a non-renal cause of hypoalbuminemia, such as suspected PLE or lupus serositis. Second, our patient had strongly positive ANA and anti-dsDNA antibodies at presentation, supporting an earlier clinical suspicion of probable SLE compared with Al-Khaldi et al.'s seronegative case. Third, our patient did not develop proteinuria during the three-month follow-up period, supporting the hypothesis of an alternative mechanism such as PLE or serositis rather than evolving nephritis. The present case adds to this limited literature by demonstrating that periorbital edema may be the sole presenting feature in adolescent SLE and that a high index of suspicion for PLE or serositis is warranted even in the absence of gastrointestinal symptoms.

Diagnostic criteria and classification

Formal application of classification criteria in this case was limited by the absence of several key diagnostic investigations. The 2019 European League Against Rheumatism/American College of Rheumatology criteria specify pleural or pericardial effusion and acute pericarditis within the serosal domain but do not include peritoneal involvement or ascites as scored items [11]. Similarly, the 2012 Systemic Lupus International Collaborating Clinics criteria define serositis as pleuritis or pericarditis, with peritoneal involvement not formally recognized [10]. Consequently, the patient did not accumulate sufficient criteria for formal classification under either system, despite a clinical phenotype strongly suggestive of probable SLE. This limitation highlights the need for increased awareness of underrecognized gastrointestinal and peritoneal manifestations of SLE, which may warrant consideration in future diagnostic frameworks as larger case series and systematic studies emerge.

Treatment response and clinical course

The prompt clinical and biochemical response to corticosteroid therapy is consistent with previous reports of PLE in SLE [6,7]. The normalization of serum albumin to 3.8 g/dL within three months, accompanied by resolution of ascites and improvement in hemoglobin and inflammatory markers, supports the hypothesis of an immune-mediated mechanism responsive to anti-inflammatory therapy.

However, we acknowledge that the observed response does not definitively establish PLE as the underlying mechanism, as lupus serositis would also be expected to respond to corticosteroids. The absence of a diagnostic biopsy or functional study remains a limitation in attributing the clinical improvement to a specific pathophysiological process.

Clinical importance

This case carries several practical implications for clinicians. First, adolescents presenting with unexplained periorbital edema should undergo systematic evaluation for autoimmune etiologies, including SLE, even in the absence of classical features such as malar rash, arthritis, or renal abnormalities. Second, the combination of hypoalbuminemia and ascites without proteinuria should prompt consideration of PLE or serositis in patients with known or suspected SLE. Third, resource-limited settings may preclude definitive diagnostic confirmation, yet clinical suspicion combined with characteristic laboratory findings and response to empiric therapy may guide management. Finally, early initiation of corticosteroid therapy appears to be associated with favorable outcomes, although long-term follow-up is necessary to assess disease remission and the potential need for steroid-sparing agents.

Periorbital edema has a broad differential diagnosis and is not specific to SLE. Other important differential diagnoses include nephrotic syndrome, chronic liver disease, hypothyroidism, allergic angioedema, orbital cellulitis, dermatomyositis, and superior vena cava obstruction. In the present case, these conditions were considered and excluded based on the patient's history, physical examination, laboratory investigations, and imaging findings. Therefore, the clinical diagnosis of probable SLE was based on the overall clinical and immunological findings rather than on periorbital edema alone.

We acknowledge that initiating corticosteroid therapy before definitive diagnostic confirmation carries potential risks, including masking alternative diagnoses and delaying appropriate treatment. In the present case, corticosteroid therapy was initiated based on the overall clinical assessment, supportive laboratory findings, exclusion of the most likely alternative diagnoses, and close clinical follow-up. Therefore, this management should be interpreted as an individualized clinical decision rather than a general recommendation for empiric corticosteroid therapy in all patients with suspected SLE.

Limitations

This case has several important limitations. Serum complement (C3 and C4) levels and the direct Coombs test were unavailable at our institution. In addition, confirmatory investigations for PLE, including fecal alpha-1 antitrypsin clearance, technetium-99m albumin scintigraphy, upper and lower gastrointestinal endoscopy with biopsy, and paracentesis with ascitic fluid analysis, could not be performed. Consequently, the diagnosis of probable SLE and suspected PLE or lupus serositis was based on clinical judgment, supportive laboratory findings, exclusion of alternative diagnoses, and response to corticosteroid therapy rather than definitive diagnostic confirmation.

## Conclusions

This case demonstrates that isolated periorbital edema may serve as the initial manifestation of probable SLE in adolescents, even in the absence of typical systemic features such as nephritis, arthritis, or dermatitis. The presence of hypoalbuminemia with ascites and normal urinary protein excretion should raise suspicion for PLE or lupus serositis in the differential diagnosis. While confirmatory testing was unavailable in this case, the characteristic clinical and laboratory findings, combined with a favorable response to corticosteroid therapy, support the clinical utility of early immunological evaluation and empiric treatment. Clinicians should maintain a broad differential when evaluating adolescents with unexplained edema, as prompt recognition and management of underlying SLE may facilitate early diagnosis and reduce the risk of organ damage. Long-term follow-up and prospective studies are needed to better characterize the natural history and optimal management of PLE in childhood-onset SLE.
